# Factors associated with small-scale agricultural machinery adoption in Bangladesh: Census findings

**DOI:** 10.1016/j.jrurstud.2016.06.012

**Published:** 2016-08

**Authors:** Khondoker Abdul Mottaleb, Timothy J. Krupnik, Olaf Erenstein

**Affiliations:** a International Maize and Wheat Improvement Center (CIMMYT), Carretera México-Veracruz Km. 45, El Batán, Texcoco C.P. 56237, Mexico; b International Maize and Wheat Improvement Centre (CIMMYT), Bangladesh Country Office, House 10/B, Road 53, Gulshan-2, Dhaka 1213, Bangladesh

**Keywords:** Farm mechanization, South Asia, Scale-appropriate machinery, Sustainable intensification, Labour scarcity, Gender equity

## Abstract

There is strong advocacy for agricultural machinery appropriate for smallholder farmers in South Asia. Such ‘scale-appropriate’ machinery can increase returns to land and labour, although the still substantial capital investment required can preclude smallholder ownership. Increasing machinery demand has resulted in relatively well-developed markets for rental services for tillage, irrigation, and post-harvest operations. Many smallholders thereby access agricultural machinery that may have otherwise been cost prohibitive to purchase through fee-for-service arrangements, though opportunity for expansion remains. To more effectively facilitate the development and investment in scale-appropriate machinery, there is a need to better understand the factors associated with agricultural machinery purchases and service provision. This paper first reviews Bangladesh’s historical policy environment that facilitated the development of agricultural machinery markets. It then uses recent Bangladesh census data from 814,058 farm households to identify variables associated with the adoption of the most common smallholder agricultural machinery – irrigation pumps, threshers, and power tillers (mainly driven by two-wheel tractors). Multinomial probit model results indicate that machinery ownership is positively associated with household assets, credit availability, electrification, and road density. These findings suggest that donors and policy makers should focus not only on short-term projects to boost machinery adoption. Rather, sustained emphasis on improving physical and civil infrastructure and services, as well as assuring credit availability, is also necessary to create an enabling environment in which the adoption of scale-appropriate farm machinery is most likely.

## Highlights

• Few farm households in developing countries can invest in agricultural machinery.
• Fee for service systems can however create broader access.
• Using 814,058 households, we characterized farmers who invest in agricultural machinery.
• Physical assets, institutional and physical infrastructure positively affect adoption.
• Policy makers and donors promoting mechanization should also emphasize these factors.

## 1 Introduction

By 2050, global population is expected to reach 9.6 billion (Gerland et al., 2014). As a result, the consumption of staple cereals, including rice, wheat, maize, as well as fish and meat products is expected to increase dramatically – particularly in rapidly developing countries (Godfray and Garnett, 2014). To ensure cereal food security alone in 2050, more than a doubling of production is required (Tilman et al., 2011). This situation is complicated by the anticipated strain on global cropland availability, resulting in calls to intensify production on available land in order to avoid natural land conversion and biodiversity loss, while also working to reduce food waste and curb overly consumptive diets (Tilman et al., 2011).

These problems are particularly acute in densely populated South Asia, which records the highest number of extremely poor people living on less than USD 1.25 day^−1^, estimated at 399 million in 2011 (World Bank, 2015b). Over half of the population depend primarily on agriculture, and average farm sizes range from just 0.53 to 2.1 ha (Hossain et al., 2007, PBS, 2010). In 1961, per capita arable land in Bangladesh, India, Nepal, and Pakistan ranged between 0.17 and 0.36 ha, though by 2012, these figures shrunk to 0.12–0.24 ha. Agriculture is also a leading source of income and employment in rural areas. In 2005, for example, 48% of the total employed labour force in Bangladesh, 56% in India, 43% in Pakistan, and 66% in Nepal were directly involved in agriculture (World Bank, 2015a). Yet at the same time, rural to urban migration is increasing with the expansion of non-farm employment opportunities, causing seasonal rural labour shortages (Zhang et al., 2014).

Within this evolving context there has been strong advocacy for agricultural machinery appropriate for farmers’ small fields and resource base, and to enhance land productivity and encourage sustained agricultural intensification (FAO, 2008, Kienzle et al., 2013). Such machinery may be of interest to smallholder farmers because of potential production cost savings and reduction in drudgery by substituting manual labour and traditional tools with efficient machineries (World Bank, 2007, Kienzle et al., 2013, Mahmud et al., 2014). Such ‘scale-appropriate’ agricultural machinery is also increasingly custom-designed to be suitable for farmers’ small and fragmented landholdings, and to facilitate the conservation of agricultural resources (Krupnik et al., 2013, Krupnik et al., 2015, Baudron et al., 2015). For example, the practices of zero and strip tillage require specialized machinery and can reduce costs by saving fuel, time and irrigation water (Erenstein and Laxmi, 2008, Fileccia, 2009, Krupnik et al., 2014). For farmers using flood irrigation, machine-aided laser levelling can provide substantial water savings (Ahmad et al., 2001), reducing pressure on groundwater reserves and energy savings for pumping, thereby enhancing gross margins by up to USD 143.5 ha^−1^ (Aryal et al., 2015, Magnan et al., 2015). But despite the resulting advocacy for ‘scale-appropriate’ mechanization, questions remain as to what factors are associated with smallholder adoption, with important implications for development programs promoting mechanization.

To respond to these questions, it is necessary to understand the characteristics of farm households that invest in farm machinery, both for their own use and to rent-out services to other farmers. This consideration is important, because in contrast to the predominant model of machinery ownership on larger-sized family farms in developed countries, in emerging economies like Bangladesh, relatively few farm households invest in their own agricultural machinery. Instead, increasing numbers of smallholder farmers’ access agricultural machinery services through custom hiring arrangements (Biggs and Justice, 2015). Studying households that own machinery can provide insights into the factors that facilitate or limit such agricultural machinery investment choices, thereby aiding development planners and policy makers, including legislators who allocate public funds, as well as national and international banks to target investments more appropriately. We are however unaware of any recent studies that examine these issues at a large scale. Using Bangladesh as a case study, this paper fills this gap by identifying the factors associated with the adoption of some of the most common agricultural machineries utilized on farms, including irrigation pumps, threshers, and power tillers (used for mechanized tillage/land preparation, generally driven by a two-wheel tractor (2WT)), using census survey data.

This case is worth investigating for several reasons. The agricultural sector (excluding fisheries) contributes 12.64% of Bangladesh’s GDP (GOB, 2015). Out of 54.1 million active labourers in Bangladesh, 25.6 million (47.3%) are engaged in agriculture (GOB, 2015). Yet evidence indicates a progressive shrinking of rural labour availability, as workers migrate to cities or abroad to engage in more remunerative employment, particularly in the garments and construction sectors (Zhang et al., 2014). Projections also indicate that rice and wheat production will need to increase by 0.4 and 2.17% year^−1^, to keep pace with the additional two million added to the population annually (Mainuddin and Kirby, 2015). At the same time, there is little scope to extend the agricultural land frontier: cropland availability in Bangladesh has declined by 68,760 ha year^−1^ (0.73%) since 1976 (Hasan et al., 2013). In other words, Bangladesh needs to produce more food from the same land, while at the same time easing farm labour requirements resulting from the country’s increasingly profitable alternative forms of employment (Zhang et al., 2014).

Appropriate farm mechanization has been emphasized as an important policy and development goal in Bangladesh (Mandal, 2002, Mandal, 2014, Zhang et al., 2014). Compared to other South Asian nations, farm machinery use has advanced considerably in Bangladesh (Justice and Biggs, 2013), particularly for land preparation, irrigation, and post-harvest activities. In 1996 there were only 0.1 million power tillers, 1.3 million pumps (including deep, shallow and surface water pumps), and 0.18 million rice-wheat threshers used in Bangladesh. By the early 2010s, these figures increased to at least 0.55 million power tillers (Ahmmed, 2014), 1.61 million pumps (BBS, 2011, BADC (Bangladesh Agricultural Development Corporation), 2013), and 0.25 million threshers. Use of irrigation pumps has been a key ingredient in Bangladesh’s current level of near rice self-sufficiency (Hossain, 2009, Mainuddin and Kirby, 2015). Study of Bangladesh could therefore provide important insights into the factors that affect the spread of scale-appropriate mechanization.

In this paper, we analyse the factors associated with agricultural machinery ownership in Bangladesh. Using farm household census data, we characterize rural farm households who invest in agricultural machinery, while also assessing the role of factors such as civil and institutional infrastructure and services. We begin with a brief review of the historical policy environment that facilitated the development of agricultural machinery markets in Bangladesh, particularly the growth of agricultural mechanization since the 1970s, focusing mainly on government policy liberalizing the farm machinery sector and its underwriting with subsidy programs. We subsequently describe the census data utilized, after which we specify our econometric models, present major findings, and explore policy implications for scale-appropriate agricultural mechanization.

## 2 Agricultural mechanization in Bangladesh

With the world’s highest population density (of countries with a substantive landmass) and highest per-capita rice consumption (172.6 kg person^−1^ year^−1^; FAOSTAT, 2015), the Government of Bangladesh (GOB) has historically encouraged agricultural intensification and mechanization as an avenue to increase production and move towards rice self-sufficiency (Mainuddin and Kirby, 2015). To facilitate this process, the GOB voluntarily reduced import restrictions and tariffs on select agricultural machineries, and developed subsidy programs to partially offset fixed costs for 2WTs, irrigation pumps, and threshers (GOB, 1999). Irrigation pumps were first introduced by the GOB in the 1960s (Ahmed, 2001). Their supply was later sustained by the private sector following the GOB’s voluntary liberalization of the machinery market and relaxation of import tariffs from 1988 to 1995 (Hossain, 2009, Gisselquist et al., 2002).

The GOB also initially promoted four-wheel tractor based mechanized tillage, which is arguably scale-inappropriate given Bangladesh’s average farm size of around 0.53 ha, which is usually further fragmented into multiple fields (Hossain et al., 2007). Such fragmentation makes demand aggregation for tillage services among farmers, and between field and-farm transport difficult to achieve with larger tractors. The GOB also first introduced centralized irrigation facilities by establishing Deep Tube Wells (DTWs) and supplying surface water Low-Lift Pumps (LLPs) to farmers on a rental basis from the Bangladesh Agricultural Development Corporation (BADC), with fuel supplied at a 75% subsidized rate until the late 1970s (Hossain, 2009). By 1978, BADC had rented out and managed a total of 9000 DTWs and 35,000 LLPs (IDE, 2012).

Public irrigation management and use of larger tractors for land preparation, however presented large logistical and financial burdens. Eight years after independence in 1979, Bangladesh undertook liberalization policies, with the GOB gradually opting out of State-led mechanization support (Gisselquist et al., 2002). BADC initiated sales to liquidate DTWs and LLPs, first to farmers’ cooperatives, and later to individual farmers, many of whom became service providers (Hossain, 2009). Privatization however only gained real momentum only after the removal of tariff and non-tariff barriers on the import of irrigation and diesel engines and tractors, policy actions which were precipitated by disaster response actions on behalf of teh Government.

On November 29, 1988, a cyclone with wind speeds of over 150 km h^−1^ hit Bangladesh (UNDRO, 1988). The cyclone took a major toll on human lives, and drastically reduced the draught oxen and water buffalo population used for land preparation. The total deficiency was estimated at approximately 5.8 million animals, equivalent to 132,000 2WTs, with significant implications for the timely planting of the subsequent rice crop (GOB, 1989). During this period, the Standardized Committee of Bangladesh was responsible for controlling the quality of imported machinery, including agricultural equipment. The committee mainly prescribed the import of high-cost Japanese tractors, pumps, and engines, and discouraged more affordable Chinese machinery that they considered to be of low quality (Justice and Biggs, 2013). The urgency imposed by the cyclone and risk of food insecurity however prompted the GOB to reconsider this arrangement, as less expensive equipment was urgently required at a large scale. In 1988, then President Hussain Muhammad Ershad voluntarily eliminated most of the major import taxes on standardized diesel engines and 2WTs. In an effort to further facilitate the rapid import of comparatively less expensive machinery from China, he also disbanded the Standards Committee, and emphasized less expensive markets for 2WTs (Justice and Biggs, 2013). Six years later, the import 2WTs was made completely duty-free (IDE, 2012).

These actions resulted in a drastic increase in small diesel engine imports for mechanized irrigation and land preparation. The number of shallow tube wells used for irrigation increased from 93,000 in 1982 to 260,000 in 1990 (IDE, 2012). Currently, more than 550,000 power tillers, the vast majority of Chinese origin, are used to prepare over 80% of Bangladesh’s cropland (Ahmmed, 2014). A total of 1.63 million tube wells and LLPs are also used to irrigate nearly 55% of all cropland (BBS, 2011, BADC (Bangladesh Agricultural Development Corporation), 2013). In 2012–13, 112 importers invested USD 35 million to import 30,771 2WTs. In the same year, USD 0.1 million of mechanical seed drills and rice transplanters were also imported, the former of which can be attached to 2WTs for direct seeding (Krupnik et al., 2013), along with USD 2.5 million worth of spare parts (GOB , 2014a). In land and capital constrained rural Bangladesh, owners of agricultural machinery also tend to work as service providers (e.g. providing mechanized land preparation and irrigation) on a fee-per-service basis to other farmers. As a result, even the smallest farm households can usually access relatively affordable machinery services through custom hiring systems (Justice and Biggs, 2013, IDE, 2012), although the factors influencing adoption and ownership of machinery remain unclear.

## 3 Materials and methods

### 3.1 Data description

**Fig. 1:**
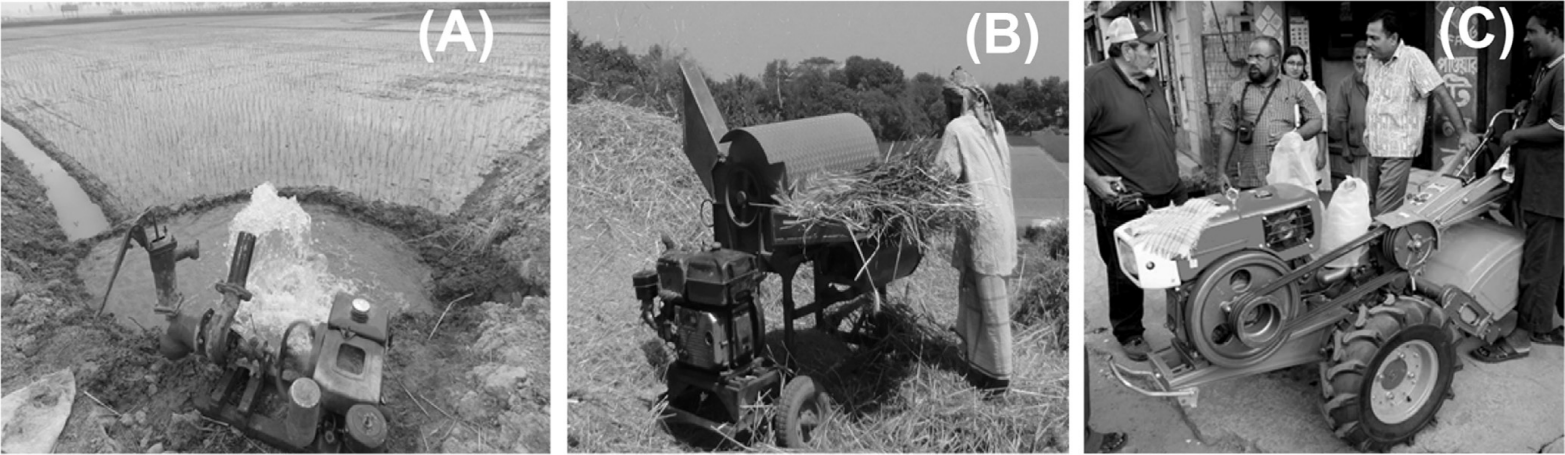
Small-scale agricultural machines considered in this study. (A) shallow tube well driven by an irrigation pump, (B) rice and wheat thresher, (C) two-wheel tractor driven power tiller.

We used two data sets from the Bangladesh Bureau of Statistics (BBS). The 2008 Agricultural Census is the fourth of its kind, and was deployed between May 11–25 of that year. The census covered all Bangladeshi farm households with a dwelling house. Data were collected using a structured questionnaire for both rural and urban areas. A total of 28.69 million farm households were surveyed, of which 25.35 million were rural from all administrative Divisions. The census provided information on ownership of the most common types of farm machinery, including irrigation pumps, threshers, and power tillers (Fig. 1). The census also provided household level information on land holdings, pond and livestock ownership, family size (segregated by adults and youth), and gender of the household head. Information on electricity availability for agricultural machinery operation, and provision of formal and informal loans, were also collected. In addition to the census data, we also collected information on the total length of paved or gravel roads at the sub-District level from the spatial data Division of Bangladesh’s Local Government Engineering Department for 2012, the most proximal year to the census for which national data were available.

**Fig. 2:**
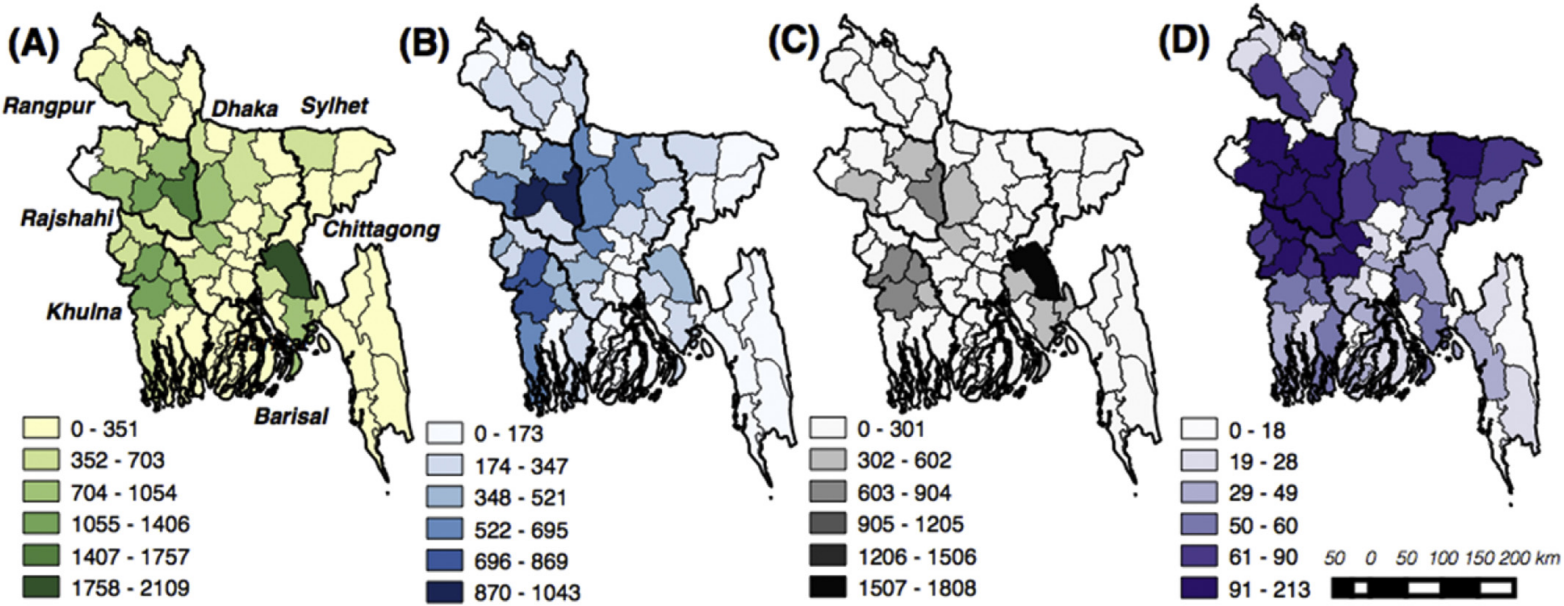
Agricultural machinery ownership by the sampled farm households in Bangladesh. Number of households reporting ownership of: (A) any of the three machine types (survey respondent owns at least one of machine types B-D), (B) irrigation pumps, (C) threshers, and (D) power tillers. Data indicate number of households in each administrative District (separated by thin black lines) reporting machinery ownership in the census survey. Names in (A) reflect administrative Divisions (thick black lines). Data source: BBS (2010).

**Fig. 3:**
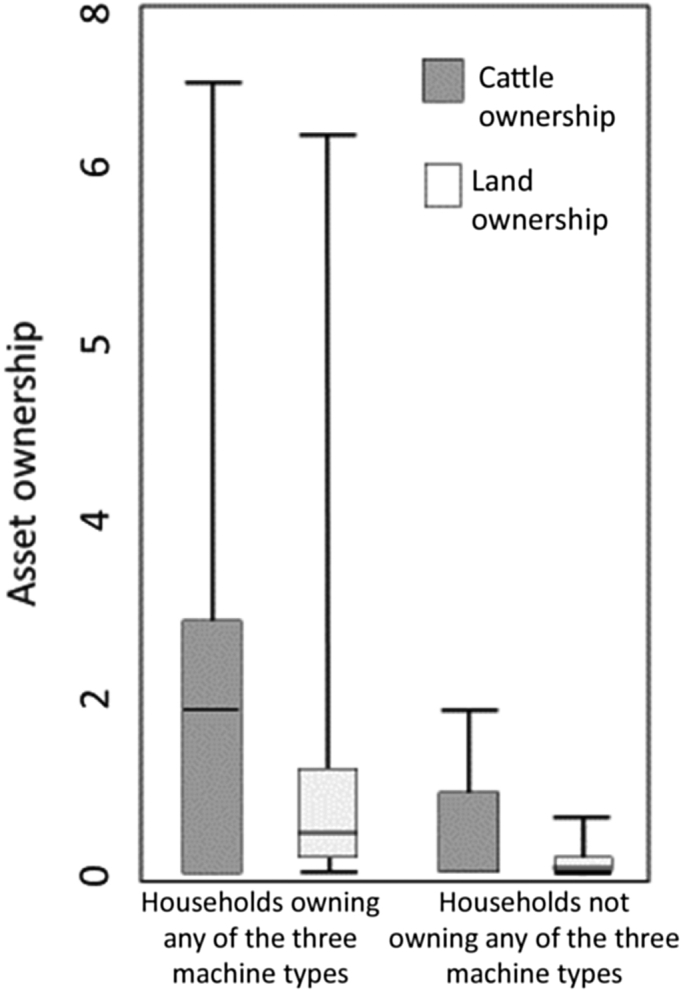
Distribution of land (ha) and cattle (no. of cows and buffalos) categorized by agricultural machine ownership by sampled rural households (HHs), excluding outliers. Source: BBS (2010).

Although the 2008 Census covered all farm households in Bangladesh, BBS provides access only to a randomly generated 5% sub-sample. This resulted in 1,163,147 households in 64 Districts in all seven Divisions of Bangladesh. Given our focus on rural areas, all urban households were excluded, resulting in 814,058 sampled households from 476 sub-Districts in all seven Divisions. Of this sample, 2%, 1.68%, and 0.45% of the households owned at least one irrigation pump, thresher, or power tiller,^1^ respectively (Table 1). Machinery ownership was uneven across the Divisions. For example, while more than 6% and 4% of households in Khulna and Rajshahi Divisions owned at least one machine type, in Barisal Division only 0.9% reported ownership (Fig. 2). Box plots illustrating the distribution of land and livestock ownership, both with and without agricultural machinery, indicated that the upper adjacent values (75th percentiles), as well as median values (mid lines) of both land and livestock ownership were higher for the households with a sampled machine compared to those without, indicating their relatively higher wealth status (Fig. 3).

### 3.2 Model specification and estimation

To examine the factors associated with the ownership of agricultural machinery in Bangladesh, we developed equation (1):

(1) $$Y_{i}=\alpha_{0}+\left(HHC_{i}\right)\emptyset +\alpha_{1}\left(Road_{sd}\right)+\alpha_{2}\left(Labor_{d}\right)+\sum_{d=1}^{6}\beta_{j}\left(DD_{j}\right)+\varepsilon_{i}$$

where *Y*_*i*_ is a vector of dependent variables including a base value of zero if a household did not own an irrigation pump, thresher, or power tiller in 2008, an pump ownership dummy assuming a value of one for ownership, a thresher ownership dummy assuming a value of two for ownership, and power tiller ownership dummy assuming a value of three.^2^ Among the explanatory factors, *HHC*_*i*_ is a vector of independent variables including a gender dummy that assumes a value of one if a household head is female (zero otherwise), a dummy that assumes a value of one if the household head does not work as an agricultural labour on other farms (zero otherwise), and the total number of adult family members >15 years old. Explanatory variables also include a pond ownership dummy (as pond-based aquaculture is an important source of income) and total number of cows and buffalos owned. In equation (1), the explanatory variable *HHC*_*i*_ also includes the total land owned by a household (ha), and a dummy that assumes a value of one if a household owned more than 1.01 ha, and zero for below this threshold.

According to the BBS (2011a), smallholder households own up to 1.00 ha, medium-sized farm households own 1.01–3.03, and large ones own >3.03 ha. Thus, the land ownership dummy in our model combines medium and large farms, capturing the effect of farm size on the ownership of agricultural machinery even after controlling for the influence of total land size owned by a household. Among the explanatory variables, *HHC*_*i*_ also includes a dummy that assumes a value of one if a household borrowed money from any formal or informal sources, or zero otherwise, and a dummy with a value of one if a household runs at least one piece of agricultural machinery using electricity (zero, otherwise). *Road*_*sd*_ is an independent variable that includes information on the length of paved or gravel roads (km) at the subDistrict level. Finally, to capture the influence of labour availability on machinery adoption, we included the independent variable *labuor*_*d*_, using District-level information on the number of households who work as agricultural labourers on other farms. By definition, these households’ major source of income was obtained by working as agriculture labourers on land owned by other farmers (BBS, 2011a). The availability of labour could differentially affect machinery ownership. For example, pumps could increase labour demand if irrigation is used to intensify and grow a dry-season crop; by contrast, power tillers and threshers may reduce labour requirements. To understand if sub-District level factors such as paved or gravel roads and loan facilities affect machinery adoption by medium and large farm households compared to small farm households, we also included two multiplicative dummies, in which we multiplied the medium and large farm household dummy with road length. We also multiplied the medium and large farm household dummy with the dummy for households borrowing funds from formal or informal sources. DD_j_ represents six Division dummies for seven administrative Divisions, with Chittagong as the base. *α*_0_ is the scalar parameter, and ϕ, *α* and β are the vectors of parameters; *i* stands for household, *sd* stands for sub-District, *d* for District, with ξ for random error.

The most common machine owned by a household is an irrigation pump; however, ownership of multiple machines is also possible (Table 1). We therefore applied a multinomial probit model, commonly used to explain farmers’ adoption of multiple technologies. Mottaleb et al. (2014), for example, used this method to estimate the use of hybrid and inbred rice on different fields of the same farm in Bangladesh, while Quayam and Ali (2012) also applied a single logit model to estimate the adoption of power tillers in selected locations of Bangladesh.

We estimated four models to assess the influence of these variables on household adoption of agricultural machinery, and to control against any potential endogeneity problems in the dataset. In the first unrestricted full model, we included all possible explanatory variables described above. We also estimated an additional three restricted models (R1-R3), in which we removed selected variables to avoid any potential endogeneity and redundancy problems, and in order to isolate the remaining variables of explanatory interest. For example, because we included the land size owned in the unrestricted model, one might argue that further inclusion of a dummy variable for medium and large farm household and allied multiplicative dummies might be redundant. In R1, we consequently removed the dummy for medium- and large sized farms, and related multiplicative dummies. In R2, we removed the pond ownership dummy (yes = 1), and the number of cows, water buffalos, and land owned by a household. We removed these variables as one may argue that a household might first invest in agricultural machinery, and later after generating income by using this machinery to provide services to other farmers, further investment in other resources such as in land or ponds may occur. In the third restricted model (R3), we removed the dummy variable for household receipt of loans to control for those which may have been awarded based the on human and physical capital of a household that could be used as collateral.

## 4 Results and discussion

### 4.1 General survey results

On average, a sampled household was endowed with 0.31 ha of land and one livestock head (cows and/or water buffalos). 24% owned a pond, and 92% were smallholders (with <1.01 ha, Table 2). Data showed that 3.4% of the sampled farm households owned at least one irrigation pump, thresher, and/or power tiller (Table 1). A smaller national-level household survey recently indicated that 72% and 66% of the region’s farmers nonetheless regularly access power tillers and pumping for irrigation through fee-for-service arrangements, though data for access to threshers were lacking (Ahmed et al., 2013). Mandal (2014) indicated that because Bangladesh’s average farm size is small, farmers who are agricultural machinery owners tend to enter into small-scale businesses to serve other farmers, in order to generate additional revenue after attending to their own fields. Such entrepreneurship is especially common where farmer-clients are consolidated in the same or nearby villages. This model has generated a large number of service providers providing access to irrigation and land preparation services, with other studies indicating similar growth for post-harvest threshing and shucking services (IDE, 2012, Quayum and Ali, 2012, Justice and Biggs, 2013, Mandal, 2014).

Compared to the national average and other Divisions, farm households in Barisal Division, located in the central southern coast, were however less likely to own agricultural machinery (Table 2). Households in Khulna, Rajshahi and Chittagong Divisions, conversely, are more likely to own machinery compared to the households in other Divisions. In Barisal, 0.9% of the households owned one of the machines inventoried in the survey: 0.28% owned an irrigation pump, 0.50% owned a thresher, and 0.24% owned a power tiller. Cropping intensity, defined as the number of crops grown per calendar year on the same piece of land, is low in Barisal (MoA and FAO, 2013), owing partly to the difficulty of establishing a dry season crop in this coastal region, where tidal flooding, seasonal cyclones, and salinity are concerns, and also due to farmers’ perception of production risks that encourage a lack of investment (Mottaleb et al., 2013, MOA, 2013). From 1960 to 2010, for example, 45 cyclones crossed Bangladesh’s coast and passed through this region (e.g., Mottaleb et al., 2013). In combination, these and other influences result in above average poverty rates in Barisal (World Bank, 2010). These factors appear to also influence farmers’ level of risk aversion (MoA and FAO, 2013), which may result in a lower willingness to invest in agricultural machinery or intensified cropping.

More than 25% of the sampled households received loans or credit services from banks or NGOs. On average, sub-Districts had 190 km of paved or gravel roads. On average, nearly 3% of the sampled households operated at least one agricultural machine using electricity, primarily for irrigation pumps that require connections for deeppumping, and to a lesser extent for shallow tube well extraction. Civil infrastructure, such as roads and electricity networks, and institutional infrastructure, such as loan facilities, could affect both the availability of agricultural machineries as well as farmers’ decisions to invest in their purchase, affecting overall operation and transaction costs for both suppliers and machinery owner-operators. Electricity availability could for example encourage a household to purchase an irrigation pump, as the availability of subsidy programs for electrically driven tube wells offers an inexpensive energy source (BIDS , 2012). Diesel driven pumps also tend to require pre-season purchases of fuel, and entail costs for transporting fuel from the purchase point to the pumping station. Similarly, the extent of accessible loan facilities could affect machinery investment decisions, especially for more costly power tillers, which average in excess of USD 1000 unit^−1^. Each Division had an average of 218 thousand households with members that worked as agricultural labourers on other farms. Such labour was however relatively scarcer in Barisal Division, while it was comparatively abundant in Rajshahi (Table 2). Less than 4% of the households included in the census data were female-headed. Overall, the average household was endowed with more than three family members, of which more than 50% were adults >15 years old (Table 3).

### 4.2 Estimated unrestricted functions

All non-multiplicative household level variables included in the estimated unrestricted function (in which we included all possible explanatory variables) were highly statistically significant at the 95 or 99% probability level, with the expected coefficient signs for explaining ownership of irrigation pumps, threshers, and power tillers (Table 4). The dummy for the household owner who never worked as a wage labourer, number of adults, pond ownership, livestock number, total land owned, and medium and large farm household size dummies were all positively and significantly (*P* < 0.001) related to machinery adoption.

Conversely, the female-headed household dummy was negative and significant (*P* < 0.001) in the estimated functions relating this variable to thresher and 2WT ownership, though there was no statistically significant difference in ownership of pumps. These results support the findings of previous research in Bangladesh showing that women headed households are less likely to own agricultural assets than those headed by men (e.g., Quisumbing et al., 2001). In addition to underlying resource differences between these household types, this may also be associated with social convention. Full use of threshers and power tillers through service provision requires frequent movement within and among villages, and negotiation with client farmers, whereas social convention in Bangladesh to some extent limits women’s movement outside the household, especially without male supervision (Jahan, 2015). Irrigation pumps represent relatively smaller capital investments and can be placed semi-permanently at tube-well locations and then operated with a fixed-client base, making ownership potentially more plausible for female-headed households. The lack of agricultural machinery ownership among women except irrigation pumps however requires further investigation, but is an important topic for policy planners and development organizations concerned with increasing gender equity.

The medium and large farm size dummy was positive and highly significant (*P* < 0.001) with ownership, indicating influence on ownership of all three machines investigated. Medium and large farm households tend to have a higher probability of having a sampled agricultural machine compared to a smallholder household, reflecting the more resource-endowed nature of larger households. This is an important point for development and policy planners that focus on extending the use of scale-appropriate machinery to smallholders, as well as to private sector investors interested in accessing smallholder markets. Where projects seek to facilitate private sector led development and business models to encourage purchase of equipment through commercial channels, as is common in value-chain and so-called ‘making markets work for the poor’, or M4P projects (DFID and SDC, 2008), our data suggest that poorer households are unlikely to be willing or are unable to invest in machinery purchase. This may not however exclude access to machinery, as projects can focus on service provision and custom hiring arrangements at fees affordable to smallholders. Experience has shown that this is the main mechanism by which farmers are able to access irrigation pumps, threshers, and power tillers in South Asia and Bangladesh (Hossain, 2009, Justice and Biggs, 2013, Mandal, 2014), underscoring that not all farmers must own a machine to expand their use. Further research into the dynamics of service provision markets, and their influence on machinery adoption is warranted, and could be addressed in the next Bangladesh agricultural household census.

The institutional level variable, the dummy for households who have received financial loans both from formal and informal sources, and the infrastructural dummy for electricity availability within sampled households were both significantly (*P* < 0.001) and positively related to the ownership of all sampled agricultural machinery in the unrestricted model (Table 4). Another infrastructural variable, the extent of paved or gravel road at the sub-District level however positively correlated with the ownership of all sampled agricultural machinery, although only significant in the case of irrigation pumps and threshers (*P* < 0.001), but not power tillers. The 2WTs primarily used to drive power tillers are versatile and can be transported easily even on difficult terrain. The power tiller attachment can also be removed, and attachable flatbed trailers can be hitched to 2WTs and used to haul people and materials prior to, or after land preparation and tillage is completed, thereby extending the use the 2WT for more days of the calendar year (Biggs and Justice, 2015). Such 2WT use provides a key mode of transport in underserved and difficult to reach areas with poor road networks. Charging for transport also provides an extra income source to repay investment in 2WTs in areas where roads and public transport are poorly developed (Justice and Biggs, 2013). This may partially explain why the extent of paved or gravel road had no significant relation to the adoption of power tillers. Our findings do not however deny that the prevalence of paved roads and highways can enhance the adoption of other agricultural machineries (Table 4), and also augment the flow of information, both between farmers and extension workers who can more easily access farmers where denser and quality road networks are prevalent (Mottaleb et al., 2014a).

The variables in which we multiplied the medium- and large-farm household dummy with the length of paved or gravel road at the sub-District level, and also with the loan facilities, further elucidates how infrastructural and institutional variables may be differently related to the adoption of agricultural machineries by differently size households. The coefficients and the corresponding standard errors (ranging from 0.01 to 0.04) in the unrestricted estimated function show that while institutional and infrastructural variables positively influence agricultural machinery ownership, these variables do not generate any positive bias by favouring medium- and large-farm households compared to smallholders. Rather, it indicates that the institutional and infrastructural variables generate almost equal influence on households irrespective of the sizes of their land holdings. Quayum and Ali (2012) also found that credit availability can significantly and positively affect the adoption of power tillers, and Mottaleb et al. (2014) demonstrated that physical infrastructure significantly influence the adoption of new agricultural technologies such as hybrid rice in Bangladesh.

The number of household members who also engaged as agricultural labourers on other farms was differentially related to the ownership of each machine studied. This variable was positively and significantly (*P* < 0.001) correlated with the ownership of irrigation pumps, although it was negatively and significantly related to the ownership of threshers and power tillers (*P* < 0.001). These negative relationships indicate that threshers and power tillers are likely, to some extent, to displace the agricultural labour. By contrast, use of irrigation has helped to increase cropping intensity in Bangladesh (Hossain, 2009), which can generate an increase in labour demand. Further research would however be necessary to substantiate whether or not threshers and power tillers do displace agricultural labour, though this is beyond the scope of this study.

### 4.3 Restricted models

In restricted model R1, the dummies for medium- and large-farm size were removed, as was the product of this dummy with the length of paved or gravel road at the sub-District level, and the dummy for loan access. In the second restricted model (R2), the dummy for pond ownership, total land area owned, and number of livestock were removed. In the third restricted model (R3), the dummy for loans taken by households was removed. After running R1 and R2, the institutional variable dummies for provision of credit services and the infrastructural variable dummy for use of machinery reliant on electricity remained highly statistically significant (*P* < 0.001) and positive (Table 4). The estimated functions in model R1 and R2 thus indicate the robustness of the relationship of credit as an institutional service variable and infrastructural facilities such as the availability of electricity at the village level on the ownership of pumps, threshers, and 2WTs. The results of R2 in particular confirm that institutional and infrastructural facilities did not generate any significant bias against agricultural machinery ownership based on the size of the farm households (with standard errors ranging between 0.01 and 0.04).

In the third restricted model R3, besides removing any remaining potentially endogenous variables, we also removed dummy for households that received loans from formal or informal sources. Similar to the estimated functions in R1 and R2, R3 also showed statistical significance (*P* < 0.001) after re-running the model, indicating the robustness of the influence of physical infrastructure such as the electricity availability the village level on the ownership of agricultural machinery at the farm household level.

To validate the estimated models, we conducted log likelihood ratio tests considering all restricted models (R1, R2 and R3) as nested within the unrestricted model. The estimated *χ*^2^ statistics are for R1: *χ*^2^ = 865.88, for R2: *χ*^2^ = 3607.51 and for R3: *χ*^2^ = 3856.13, with 1% level of significance suggested that the unrestricted model (in which all restricted models are nested) in predicting the ownership of sampled agricultural machinery is more acceptable than the restricted models, confirming its robustness (Table 4).

Our dataset consisted of 814,058 farm households. The large size of the dataset could arguably generate significant results simply as a result of the statistical power accrued from the sample size. We therefore also examined the sensitivity of our results by estimating additional functions for the same variables as Table 4, by randomly bifurcating the data into 75% (*n* = 610,543) and 25% sub-populations (*n* = 203,515) and re-running all models (Annex 1 and 2). No changes in the signs of the relationships between variables were found after analysing the bifurcated data. Significance levels changed in just 0–2% and 1–2% of the cases considering the 75% and 25% population bifurcations across the unrestricted and restricted models, respectively. These results further indicate that our models appear to be relatively robust to changes in the sample size considered.

### 4.4 Regional heterogeneity in machinery ownership

Using Chittagong Division as a baseline, the extent of adoption of agricultural machinery in Barisal and Sylhet Divisions, which are located on the south central coast and northeast of Bangladesh, respectively, is low in comparison to other Divisions (Fig. 2). To compare the heterogeneity in machinery adoption at the Division level, we therefore treated Chittagong Division as a base in our estimated functions in Table 4. Approximately 65% of Chittagong’s land area is located in mountainous or hilly environments where forest cover predominates. This area has very deep water tables, and low agricultural coverage and cropping intensity (MoA and FAO, 2013). Machinery use is therefore low in Chittagong, and as such we selected it to contrast with other Divisions and to provide a conservative estimate of mechanization potential.

The challenges to increased agricultural productivity in Barisal Division include an above average prevalence of poverty (World Bank, 2010), mounting soil salinity in the coastal fringe, regular tidal flooding, and increased risk of impact by cyclonic storms. Out-migration by agricultural labourers is also common (MoA and FAO, 2013). One might expect that out-migration would encourage agricultural machinery ownership, in order to offset seasonal labour deficits, although our results provided no indication of increased machinery ownership or use. The prevalence of risk, biophysical production constraints, and farmers’ limited investment capacity, in addition to electrification rates below than the national average (BBS, 2010), appear to be related to the limited uptake of machinery in Barisal.

These unique circumstances therefore suggest that the GOB and donors may wish to consider special programmes to encourage scale-appropriate mechanization in Barisal. This Division has already been targeted for the expansion of surface water irrigation initiatives to increase cropping intensity (MoA and FAO, 2013), with studies indicating that use of low-lift pumps and/or mechanized land preparation could assist in moving farmers from single to double cropping where both irrigated and rainfed agriculture is practiced during the winter season, respectively (Krupnik et al., 2014, Krupnik et al., 2015, Schulthess et al., 2015). Abiotic stress resistant varieties and improved agronomic practices could be useful in this regard (Mottaleb et al., 2013). We however underscore that sufficient attention must also be placed on the development of infrastructural facilities, and to the mitigation of agricultural investment risk – which could be partially ameliorated through low-interest and low risk credit or viable crop insurance programs, though recent evidence in Bangladesh indicates the importance of careful insurance design and efforts to assure that smallholders understand and see benefit from these mechanisms (e.g., Akter et al., 2016). Rather than focus on immediate interventions to encourage farmers to secure machinery, it may be more logical to sequence them by concentrating on civil and instructional infrastructural development as prerequisite to create an enabling and reduced-risk environment in which machinery value chains can be created and adoption can be accelerated. Conversely, efforts to generate an enabling environment by developing infrastructure could be funded simultaneously with programs facilitating for machinery adoption, although our data indicate that a sufficient level of infrastructural development is likely to be an initial prerequisite.

## 5 Conclusion

Scale-appropriate agricultural mechanization can play an important role in enhancing the labour productivity of smallholder farmers. A better understanding of the socioeconomic factors that influence farmers’ ability to purchase and adopt small-scale agricultural machineries in South Asia helps inform policy. Service provision and fee-for-use models extend machinery access to farmers lacking sufficient capital for actual machinery purchase. Such understanding is crucial for scaling-out appropriate agricultural machinery within South Asia and other developing regions. Based on our review of the literature, relatively little research has considered these issues, and no prior empirical studies have employed data at the scale considered in this paper, with 814,058 observations to examine household characteristics, socio-economic, and infrastructural variables as they relate to machinery ownership. Following a brief review of Bangladesh’s historical policy environment that facilitated the development of agricultural machinery markets, this paper presents a first-step to fill this knowledge gap by identifying some of the factors that influence the ownership of the three most common types of agricultural machineries in our case study country, namely irrigation pumps, grain threshers, and power tillers.

The wealth status and land size holding of the sampled households was significantly and positively related to the ownership of agricultural machinery at the household level. Those households that were endowed with more land, cattle and ponds, were significantly more likely to have adopted and own agricultural machinery – supporting expectations that capital good ownership is likely to be positively associated with the owners’ overall resource base. Our data also indicate that civil infrastructure – primarily the availability of electricity (specifically for irrigation pumps) – and access to credit services, are both significantly and positively related to household ownership of agricultural machinery. Paved or gravel roads at the sub-District level- were also significantly and positively related with ownership of irrigation pumps and threshers, though not power tillers. The lack of influence observed here is most likely because the 2WTs primarily used to drive power tillers are relatively all-terrain vehicles, and can easily be moved along village paths and unpaved roads. Conversely for irrigation pumps, road networks are important for assuring that surplus harvests in irrigated systems can be efficiently brought to markets; while for threshers, quality roads are usually required to move equipment to consolidated threshing points, after which grain is bulked and carried to markets. Lastly, when farmers had improved access to credit and loans, either through formal banks or NGOs, machinery ownership was significantly more common. These findings indicate that the provision of basic civil infrastructure and services in Bangladesh’s rural areas appear to be prerequisites to irrigation pump, thresher, and power tiller ownership by farm households, and by consequence to the development of rural agricultural machinery service provision economies, although these data should be verified and examined for changes over time with the availability of the next agricultural census data set (anticipated in 2018). We also advise that data related to service provision arrangements for each machine should be included in future agricultural censuses, as should assessment of adoption of new scale-appropriate machineries such as modular crop reapers and planters that can be operated by 2WTs and which can help increase cropping intensity by reducing turn-around time between crops. Measures to lower farmers’ production risks and provide credit services should also be considered as part-and-parcel of mechanization efforts. We conclude that development agents with an interest in expanding farmer uptake of agricultural machineries should equally consider facilitating the necessary pre-conditions to build an enabling environment for machinery ownership, and thereby encourage adoption and uptake and enhance the overall efficiency of donor investments.

## Figures and Tables

**Annex 1:** Estimated functions applying multinomial probit model explaining ownership of irrigation pumps, threshers and power tillers by 75% of the sampled farm households.

Model specification	Unrestricted full model			Restricted model R1			Restricted model R2			Restricted model R3		
Dependent variable	Pump	Thresher	Power tiller	Pump	Thresher	Power tiller	Pump	Thresher	Power tiller	Pump	Thresher	Power tiller
Female-headed household dummy (yes = 1)	−0.14*(0.07)	−0.23***(0.05)	−0.25***(0.08)	−0.14**(0.07)	−0.23***(0.05)	0.28***(0.09)	−0.15**(0.07)	−0.24***(0.05)	−0.26***(0.08)	−0.16**(0.07)	−0.25***(0.05)	−0.27***(0.08)
Dummy for machine owner-operators not employed as agricultural labourers	0.20***(0.02)	0.08***(0.02)	0.15***(0.03)	0.23***(0.02)	0.07***(0.02)	−0.21***(0.03)	0.22***(0.02)	0.09***(0.02)	0.17***(0.03)	0.21***(0.02)	0.08***(0.02)	0.16***(0.03)
Number of adult family member (>15 years)	0.08***(0.01)	0.11***(0.01)	0.11***(0.01)	0.09***(0.01)	0.12***(0.01)	0.13***(0.01)	0.09***(0.01)	0.14***(0.01)	0.14***(0.01)	0.097***(0.01)	0.14***(0.01)	0.14***(0.01)
Pond ownership dummy (yes = 1)	0.34***(0.02)	0.48***(0.02)	0.36***(0.02)	0.36***(0.02)	0.50***(0.02)	0.42***(0.02)						
Number of cows and buffalos	0.05***(0.00)	0.07***(0.00)	0.07***(0.00)	0.05***(0.00)	0.08***(0.00)	0.07***(0.00)						
Land owned (hectares)	0.02***(0.00)	0.03***(0.00)	0.05***(0.00)	0.05***(0.00)	0.06***(0.00)	0.08***(0.00)						
Medium- and large-farm size dummy (>1 ha) yes = 1	0.45***(0.06)	0.20***(0.04)	0.52***(0.06)				0.66***(0.05)	0.51***(0.04)	0.95***(0.05)	0.68***(0.05)	0.50***(0.04)	0.99***(0.05)
Dummy for loan and credit access (yes = 1)	0.13***(0.02)	0.16***(0.02)	0.13***(0.03)	0.13***(0.02)	0.15***(0.02)	0.16***(0.02)	0.14***(0.02)	0.18***(0.02)	0.15***(0.03)			
Dummy for agricultural machinery run using electricity (yes = 1)	4.40***(0.02)	3.20***(0.02)	3.05***(0.02)	4.41***(0.02)	3.21***(0.02)	3.09***(0.02)	4.44***(0.02)	3.26***(0.02)	3.11***(0.02)	4.44***(0.02)	3.26***(0.02)	3.12***(0.02)
Km (‘000) of paved or gravel road at the sub-District level	0.05***(0.01)	0.03***(0.01)	0.02(0.02)	0.03***(0.01)	0.04***(0.01)	0.01(0.01)	0.05***(0.01)	0.03***(0.01)	0.02(0.01)	0.05***(0.01)	0.023***(0.01)	0.01(0.01)
Km (‘000) of paved or gravel road at the sub-District level × medium and large-farm size dummy	−0.03(0.02)	0.11***(0.02)	0.03(0.02)				−0.02(0.02)	0.10***(0.02)	0.01(0.02)	−0.029(0.02)	0.10***(0.02)	0.004(0.02)
Dummy for loan and credit access × medium and large-farm size dummy	0.03(0.05)	−0.05(0.04)	0.09*(0.05)				0.03(0.05)	−0.05(0.04)	0.07(0.05)			
No. of households worked as agriculture labour on farms operated by others at the District level (‘000)	0.0004***(0.00)	−0.0002***(0.00)	−0.001***(0.00)	0.0004***(0.00)	−0.0002***(0.00)	−0.001***(0.00)	0.0004***(0.00)	−0.0002***(0.00)	−0.001***(0.00)	0.0004***(0.00)	−0.0002***(0.00)	−0.001***(0.00)
Barisal Division dummy	−0.92***(0.09)	−1.21***(0.04)	−0.33***(0.06)	−0.92***(0.09)	−1.19***(0.04)	−0.31***(0.06)	−0.84***(0.09)	−1.09***(0.04)	−0.20***(0.06)	−0.80***(0.09)	−1.05***(0.04)	−0.17***(0.06)
Khulna Division dummy	0.44***(0.04)	−0.08***(0.02)	0.27***(0.04)	0.47***(0.04)	−0.04*(0.02)	0.34***(0.04)	0.38***(0.04)	−0.16***(0.02)	0.23***(0.04)	0.41***(0.04)	−0.12***(0.02)	0.26***(0.04)
Dhaka Division dummy	0.30***(0.04)	−0.77***(0.02)	0.09**(0.04)	0.32***(0.04)	−0.75***(0.02)	0.14***(0.04)	0.19***(0.03)	−0.93***(0.02)	−0.02(0.04)	0.20***(0.03)	−0.91***(0.02)	−0.0045(0.04)
Sylhet Division dummy	−0.36***(0.06)	−1.09***(0.04)	−0.11**(0.05)	−0.34***(0.06)	−1.07***(0.04)	−0.07(0.06)	−0.35***(0.06)	−1.06***(0.04)	−0.04(0.05)	−0.35***(0.06)	−1.05***(0.04)	−0.034(0.05)
Rajshahi Division dummy	0.26***(0.04)	−0.34***(0.02)	0.40***(0.04)	0.28***(0.04)	−0.31***(0.02)	0.47***(0.04)	0.14***(0.04)	−0.51***(0.02)	0.29***(0.04)	0.15***(0.04)	−0.50***(0.02)	0.30***(0.04)
Rangpur Division dummy	0.23***(0.04)	−0.85***(0.04)	0.081(0.05)	0.26***(0.04)	−0.82***(0.04)	0.15***(0.05)	0.14***(0.04)	−0.98***(0.03)	0.01(0.05)	0.15***(0.04)	−0.97***(0.03)	0.023(0.05)
Constant	−5.00***(0.09)	−3.74***(0.05)	−4.92***(0.10)	−4.98***(0.09)	−3.80***(0.05)	−4.95***(0.10)	−4.83***(0.09)	−3.49***(0.05)	−4.73***(0.10)	−4.80***(0.09)	−3.45***(0.05)	−4.71***(0.10)
*No. of households*	610,543			610,543			610,543			610,543		
*Wald χ*^*2*^*(57)*	68080.81			68583.99			68613.41			68663.55		
*Probability* > *χ*^*2*^	0.00			0.00			0.00			0.00		
*Log pseudo likelihood*	−61163.304			−61489.551			−62536.071			−62634.341		

Notes: Numbers in parentheses are robust standard errors.

*Significant at the 10% level. **Significant at the 5% level. ***Significant at the 1% level.

**Annex 2:** Estimated functions applying multinomial probit model explaining ownership of irrigation pumps, threshers and power tillers by 25% of the sampled farm households.

Model specification	Unrestricted full model			Restricted model R1			Restricted model R2			Restricted model R3		
Dependent variable	Pump	Thresher	Power tiller	Pump	Thresher	Power tiller	Pump	Thresher	Power tiller	Pump	Thresher	Power tiller
Female-headed household dummy (yes = 1)	−0.09(0.10)	−0.07(0.08)	−0.50***(0.19)	0.08(0.10)	−0.07(0.07)	−0.51***(0.19)	−0.05(0.10)	−0.09(0.07)	−0.56***(0.19)	−0.04(0.10)	−0.11(0.07)	−0.56***(0.19)
Dummy for machine owner-operators not employed as agricultural labourers	0.23***(0.04)	0.08***(0.03)	0.14***(0.04)	0.25***(0.04)	0.10***(0.03)	0.19***(0.04)	0.25***(0.04)	0.09***(0.03)	0.17***(0.04)	−0.24***(0.04)	0.08***(0.03)	0.15***(0.04)
Number of adult family member (>15 years)	0.06***(0.01)	0.13***(0.01)	0.11***(0.01)	0.02***(0.01)	0.14***(0.01)	0.13***(0.01)	0.09***(0.01)	0.16***(0.01)	0.15***(0.01)	0.09***(0.01)	0.16***(0.01)	0.15***(0.01)
Pond ownership dummy (yes = 1)	0.31***(0.04)	0.46***(0.03)	0.36***(0.04)	0.33***(0.04)	0.49***(0.03)	0.41***(0.04)						
Number of cows and buffalos	0.07***(0.01)	0.08***(0.01)	0.09***(0.01)	0.08***(0.01)	0.09***(0.01)	0.091***(0.01)						
Land owned (hectares)	0.03***(0.01)	0.03***(0.01)	0.05***(0.01)	0.05***(0.01)	0.06***(0.00)	0.082***(0.01)						
Medium- and large-farm size dummy (>1 ha) yes = 1	0.37***(0.10)	0.19**(0.08)	0.50***(0.10)				0.64***(0.09)	0.50***(0.07)	0.99***(0.09)	0.64***(0.08)	0.46***(0.06)	0.96***(0.09)
Dummy for loan and credit access (yes = 1)	0.12***(0.04)	0.22***(0.03)	0.15***(0.05)	0.12***(0.04)	0.18***(0.03)	0.11***(0.04)	0.14***(0.04)	0.24***(0.03)	0.17***(0.05)			
Dummy for agricultural machinery run using electricity (yes = 1)	4.32***(0.04)	3.10***(0.03)	3.01***(0.04)	4.33***(0.04)	3.12***(0.03)	3.05***(0.04)	4.37***(0.04)	3.17***(0.03)	3.07***(0.04)	4.38***(0.04)	3.17***(0.03)	3.08***(0.04)
Km (‘000) of paved or gravel road at the sub-District level	0.06***(0.02)	0.04***(0.01)	−0.01(0.03)	0.04**(0.02)	0.06***(0.01)	−0.013(0.02)	0.05**(0.02)	0.04***(0.01)	−0.010(0.03)	0.05**(0.02)	0.033**(0.01)	−0.02(0.03)
Km (‘000) of paved or gravel road at the sub-District level × medium and large-farm size dummy	−0.01(0.04)	0.13***(0.03)	0.04(0.04)				−0.013(0.04)	0.13***(0.03)	0.020(0.04)	−0.02(0.04)	0.13***(0.03)	0.020(0.04)
Dummy for loan and credit access × medium and large-farm size dummy	−0.02(0.08)	−0.17**(0.07)	−0.11(0.09)				−0.02(0.08)	−0.16**(0.07)	−0.11(0.09)			
No. of households worked as agriculture labour on farms operated by others at the District level (‘000)	0.001***(0.00)	−0.0001(0.00)	−0.001***(0.00)	0.001***(0.00)	−0.00004(0.00)	−0.001***(0.00)	0.001***(0.00)	−0.0001*(0.00)	−0.001***(0.00)	0.001***(0.00)	−0.0001(0.00)	−0.001***(0.00)
Barisal Division dummy	−0.77***(0.16)	−1.27***(0.08)	−0.42***(0.11)	−0.77***(0.16)	−1.26***(0.08)	−0.41***(0.11)	−0.67***(0.16)	−1.15***(0.08)	−0.26**(0.10)	−0.64***(0.16)	−1.10***(0.08)	−0.23**(0.10)
Khulna Division dummy	0.38***(0.06)	−0.08**(0.04)	0.24***(0.07)	0.40***(0.06)	−0.03(0.04)	0.30***(0.07)	0.34***(0.06)	−0.15***(0.04)	0.21***(0.07)	0.37***(0.06)	−0.11***(0.04)	0.24***(0.07)
Dhaka Division dummy	0.31***(0.06)	−0.73***(0.04)	0.021(0.07)	0.32***(0.06)	−0.70***(0.04)	0.06(0.07)	0.21***(0.06)	−0.88***(0.04)	−0.078(0.06)	0.23***(0.06)	−0.85***(0.04)	−0.07(0.06)
Sylhet Division dummy	−0.31***(0.11)	−1.18***(0.07)	−0.10(0.09)	−0.30***(0.11)	−1.17***(0.07)	−0.08(0.09)	−0.26**(0.11)	−1.14***(0.07)	0.004(0.09)	−0.26**(0.10)	−1.12***(0.07)	0.01(0.09)
Rajshahi Division dummy	0.26***(0.06)	−0.36***(0.04)	0.41***(0.07)	0.28***(0.06)	−0.32***(0.04)	0.47***(0.07)	0.16***(0.06)	−0.53***(0.04)	0.30***(0.06)	0.17***(0.06)	−0.51***(0.03)	0.31***(0.06)
Rangpur Division dummy	0.21***(0.08)	−0.78***(0.06)	0.09(0.09)	0.23***(0.08)	−0.75***(0.06)	0.14(0.09)	0.14*(0.07)	−0.90***(0.06)	0.04(0.09)	0.15**(0.07)	−0.88***(0.06)	0.04(0.09)
Constant	−4.78***(0.13)	−3.71***(0.08)	−5.11***(0.21)	−4.77***(0.12)		−5.11***(0.21)	−4.63***(0.12)	−3.46***(0.08)	−4.95***(0.20)	−4.61***(0.12)	−3.42***(0.08)	−4.92***(0.20)
*No. of households*	203,515			203,515			203,515			203,515		
*Wald χ*^*2*^*(57)*	22596.20			22383.17			22678.88			22705.27		
*Probability* > *χ*^*2*^	0.00			0.00			0.00			0.00		
*Log pseudo likelihood*	−20235.62			−20343.62			−20668.78			−20699.56		

Notes: Numbers in parentheses are robust standard errors.

*Significant at the 10% level. **Significant at the 5% level. ***Significant at the 1% level.

**Table 1:** Number of sampled farm households and their ownership of agricultural machinery types by Division in Bangladesh, 2008.

	Division							All
Barisal	Chittagong	Dhaka	Khulna	Rajshahi	Rangpur	Sylhet
Number of farm households	57,727	14,7116	24,1069	100,414	138,855	73,248	55,629	814,058
Percent owning any of the 3 machine types^a^	0.90	3.50	2.64	6.57	4.30	2.60	1.82	3.38
Percent owning an irrigation pump^b^	0.28	0.98	1.96	4.33	3.05	2.05	0.99	2.08
Percent owning a thresher^c^	0.50	2.61	0.87	3.55	2.06	0.76	0.80	1.68
Percent owning a power tiller^d^	0.24	0.23	0.39	0.66	0.74	0.40	0.51	0.45

Source: BBS (2010).

a Irrigation pump, thresher or power tiller.

b Irrigation pump includes deep tube wells, shallow tube wells, and low-lift pumps.

c Thresher includes all types of threshers, including open and closed drum threshers.

d The term power tiller is synonymous for implements used for mechanized land preparation either by a 2WT or 4-wheel tractor (4WT). In Bangladesh, power tillers are primarily associated with 2WTs with only 643 4WTs reported in the sample. The overall 0.45% power tiller ownership in the sample is the sum of 0.37% 2WT, 0.07% 4WT, and 0.01% both 2WT and 4WT.

**Table 2:** Resource endowments of sampled households by Division in Bangladesh, 2008.

	Division							All
Barisal	Chittagong	Dhaka	Khulna	Rajshahi	Rangpur	Sylhet
Land owned (ha farm^−1^)	0.37	0.27	0.29	0.36	0.30	0.24	0.43	0.31
Farm size category (% of farms)								
- Small-farm (<1.01 ha)	90.71	93.74	92.53	89.82	91.88	93.49	88.05	91.95
- Medium-farm (1.01–3.03 ha)	8.02	5.52	6.61	8.91	7.03	5.37	9.58	6.96
- Large-farm (>3.03 ha)	1.27	0.74	0.86	1.27	1.09	1.14	2.36	1.09
Cattle (cows and buffalos) owned (Number farm^−1^)	1.06	0.64	0.75	0.99	0.88	1.06	1.17	0.86
Pond ownership (% of farms)	58.57	45.27	13.46	25.50	8.39	7.12	34.58	23.90
Percent receiving loans	34.37	17.12	24.97	32.90	25.69	25.73	21.43	25.15
Percent using electricity to run machines	0.82	1.70	2.79	5.39	4.04	2.89	1.82	2.93
Paved or gravel road (sub-District level, ‘000 km)^a^	2.08	1.96	1.72	2.72	1.94	1.32	1.53	1.90
Number of households with members working as labourers on other farms District level (‘000)	91.37	220.95	167.71	159.12	201.45	217.75	124.35	178.15

Sources: BBS (2010), and GOB (2014a).

**Table 3:** Demographic characteristics of sampled households by Division in Bangladesh, 2008.

	Division							All
Barisal	Chittagong	Dhaka	Khulna	Rajshahi	Rangpur	Sylhet
Percent female-headed household	2.66	5.46	3.39	2.7	2.44	3.44	4.81	3.57
Percent adult family members^a^	51.07	47.88	50.81	50.80	53.02	50.0	45.11	50.31
Total household family members	3.27	3.78	3.09	2.81	2.81	2.76	3.99	3.18

a Indicates family members above 15 years of age.

**Table 4:** Estimated functions applying multinomial probit model explaining ownership of irrigation pumps, threshers and power tillers in Bangladesh.

Model specification	Unrestricted full model			Restricted model (R1)			Restricted model(R2)			Restricted model (R3)		
Dependent variable	Pump	Thresher	Power tiller	Pump	Thresher	Power tiller	Pump	Thresher	Power tiller	Pump	Thresher	Power tiller
Female-headed household dummy (yes = 1)	−0.07(0.06)	−0.19***(0.04)	−0.30***(0.08)	−0.08(0.06)	−0.19*** (0.04)	−0.32***(0.08)	−0.09(0.06)	−0.21***(0.04)	−0.32***(0.08)	−0.09*(0.06)	−0.21***(0.04)	−0.33***(0.08)
Dummy for machine owner-operators not employed as agricultural labourers	0.21***(0.02)	0.08***(0.01)	0.15***(0.02)	0.24***(0.02)	0.09***(0.01)	0.21***(0.02)	0.22***(0.02)	0.09***(0.01)	0.17***(0.02)	0.21***(0.02)	0.08***(0.01)	0.16***(0.02)
Number of adult family member (>15 years)	0.07***(0.01)	0.12***(0.01)	0.11***(0.01)	0.08***(0.01)	0.12***(0.00)	0.13***(0.01)	0.09***(0.01)	0.15***(0.01)	0.14***(0.01)	0.09***(0.01)	0.15***(0.01)	0.15***(0.01)
Pond ownership dummy (yes = 1)	0.33***(0.02)	0.47***(0.01)	0.36***(0.02)	0.36***(0.02)	0.50***(0.01)	0.42***(0.02)						
Number of cows and buffalos	0.06***(0.00)	0.08***(0.00)	0.07***(0.00)	0.06***(0.00)	0.08***(0.00)	0.08***(0.00)						
Land owned (hectares)	0.02***(0.00)	0.03***(0.00)	0.05***(0.00)	0.05***(0.00)	0.06***(0.00)	0.08***(0.00)						
Medium- and large-farm size dummy (>1 ha) yes = 1	0.43***(0.05)	0.19***(0.04)	0.52***(0.05)				0.66***(0.05)	0.51***(0.03)	0.96***(0.05)	0.67***(0.04)	0.49***(0.03)	0.98***(0.04)
Dummy for loan and credit access (yes = 1)	0.13***(0.02)	0.18***(0.01)	0.14***(0.03)	0.13***(0.02)	0.15***(0.01)	0.15***(0.02)	0.14***(0.02)	0.20***(0.01)	0.15***(0.03)			
Dummy for agricultural machinery run using electricity (yes = 1)	4.37***(0.02)	3.17***(0.02)	3.04***(0.02)	4.39***(0.02)	3.19***(0.02)	3.08***(0.02)	4.42***(0.02)	3.23***(0.02)	3.10***(0.02)	4.43***(0.02)	3.24***(0.02)	3.11***(0.02)
Km (‘000) of paved or gravel road at the sub-District level	0.05***(0.01)	0.03***(0.01)	0.01(0.01)	0.04***(0.01)	0.05***(0.01)	0.01(0.01)	0.05***(0.01)	0.03***(0.01)	0.01(0.01)	0.05***(0.01)	0.03***(0.01)	0.003(0.01)
Km (‘000) of paved or gravel road at the sub-District level × medium and large-farm size dummy	−0.02(0.02)	0.11***(0.01)	0.03(0.02)				−0.02(0.02)	0.11***(0.01)	0.01(0.02)	−0.03(0.02)	0.11***(0.01)	0.01(0.02)
Dummy for loan and credit access × medium and large-farm size dummy	0.01(0.04)	−0.08**(0.03)	0.04(0.04)				0.01(0.04)	−0.08**(0.03)	0.03(0.04)			
No. of households worked as agriculture labour on farms operated by others at the District level (‘000)	0.001***(0.00)	−0.0001***(0.00)	−0.001***(0.00)	0.001***(0.00)	−0.0001***(0.00)	−0.001***(0.00)	0.001***(0.00)	−0.0002***(0.00)	−0.001***(0.00)	0.001***(0.00)	−0.0002***(0.00)	−0.001***(0.00)
Barisal Division dummy	−0.88***(0.08)	−1.22***(0.04)	−0.35***(0.05)	−0.88***(0.07)	−1.21***(0.04)	−0.34***(0.05)	−0.79***(0.08)	−1.10***(0.04)	−0.22***(0.05)	−0.76***(0.08)	−1.06***(0.04)	−0.18***(0.05)
Khulna Division dummy	0.43***(0.03)	−0.08***(0.02)	0.27***(0.04)	0.45***(0.03)	−0.04*(0.02)	0.33***(0.04)	0.38***(0.03)	−0.15***(0.02)	0.23***(0.04)	0.40***(0.03)	−0.12***(0.02)	0.26***(0.04)
Dhaka Division dummy	0.30***(0.03)	−0.76***(0.02)	0.07**(0.03)	0.32***(0.03)	−0.74***(0.02)	0.11***(0.03)	0.19***(0.03)	−0.91***(0.02)	−0.034(0.03)	0.21***(0.03)	−0.90***(0.02)	−0.02(0.03)
Sylhet Division dummy	−0.35***(0.05)	−1.11***(0.03)	−0.11**(0.05)	−0.33***(0.05)	−1.09***(0.03)	−0.07(0.05)	−0.33***(0.05)	−1.08***(0.03)	−0.03(0.04)	−0.32***(0.05)	−1.07***(0.03)	−0.02(0.04)
Rajshahi Division dummy	0.26***(0.03)	−0.35***(0.02)	0.40***(0.04)	0.28***(0.03)	−0.31***(0.02)	0.47***(0.03)	0.14***(0.03)	−0.52***(0.02)	0.29***(0.03)	0.15***(0.03)	−0.50***(0.02)	0.30***(0.03)
Rangpur Division dummy	0.23***(0.04)	−0.83***(0.03)	0.09*(0.05)	0.25***(0.04)	−0.81***(0.03)	0.15***(0.04)	0.14***(0.04)	−0.96***(0.03)	0.02(0.04)	0.15***(0.04)	−0.94***(0.03)	0.03(0.04)
Constant	−4.94***(0.07)	−3.73***(0.04)	−4.95***(0.09)	−4.92***(0.07)	−3.79***(0.04)	−4.97***(0.09)	−4.77***(0.07)	−3.48***(0.04)	−4.77***(0.09)	−4.75***(0.07)	−3.44***(0.04)	−4.74***(0.09)
*No. of households*	814,058			814,058			814,058			814,058		
*Wald χ*^*2*^*(57)*	902, 00.53			910, 96.50			911, 18.19			912, 14.98		
*Probability* > *χ*^*2*^	0.00			0.00			0.00			0.00		
*Log pseudo likelihood*	−814, 30.37			−818, 63.31			−832, 34.13			−833, 58.44		
*Log likelihood ratio (LR)* (*χ*^*2*^(9))	865.88^a^						3607.51^b^			3856.13^c^		
*Prob* > *χ*^*2*^	0.00						0.00			0.00		

Notes: Numbers in parentheses are robust standard errors.

**Significant at the 5% level. ***Significant at the 1% level.

a Presents log likelihood ratio (LR) test statistics between full (unrestricted) and restricted R1 model.

b Presents log likelihood ratio (LR) test statistics between full (unrestricted) and restricted R2 model.

c Presents log likelihood ratio (LR) test statistics between full (unrestricted) and restricted R3 model.

## Footnotes

1 The term power tiller is synonymous for implements used for mechanized land preparation either by a 2WT or 4-wheel tractor (4WT). In Bangladesh, however, power tillage is primarily associated with 2WTs.

2 Our database indicated that there is limited combined ownership of three machines (Table 1). Given the agricultural machinery types, the coding assumes thresher to override irrigation pump ownership, and power tiller ownership to overrides both pump and thresher ownership (e.g., a power tiller owner who also has a thresher is classified as a power tiller owner in the model).
